# Place inclusion for older LGBT+ people and older people with Learning Disabilities: UK organisational perspectives

**DOI:** 10.1093/geroni/igaf122.145

**Published:** 2025-12-31

**Authors:** Judith Sixsmith, Mei Lan Fang, Meiko Makita, Sallie Johnson, Laura Roe, Romel Yanez

**Affiliations:** University of Dundee, Dundee, Scotland, United Kingdom; Simon Fraser University, Vancouver, British Columbia, Canada; University of Dundee, Dundee, Scotland, United Kingdom; University of Hertfordshire, Hatfield, England, United Kingdom; University of Dundee, Dundee, Scotland, United Kingdom; Federal University of Ceara, Fortaleza, Ceara, Brazil

## Abstract

Older LGBT+ individuals and those with Learning Disabilities (LD) have lived through eras of systemic stigma, exclusion, and social devaluation. The 46-month IncludeAge project (www.IncludeAge), funded by the UK Economic and Social Research Council, aims to examine the experiences of inclusion and exclusion among these groups and identify strategies to create more inclusive communities. By exploring both lived experiences and organizational perspectives, the project seeks to develop co-created solutions that enhance inclusion in place, space, policy, and practice. Eighty semi-structured interviews were conducted with advocacy and support groups for older LGBT+ and LD communities, age-related organizations, and third spaces such as churches, shops, hospitality, and welfare services. A thematic analysis of barriers and facilitators of inclusion across organizations in Scotland, England, and Wales was undertaken. Four key themes emerged as barriers to inclusion: (1) historical distrust and past negative experiences, (2) organizational limitations and financial constraints, (3) passive inclusion and limited visibility, and (4) digital (in)accessibility. Five themes were identified as facilitators: (1) communication and social networks, (2) external oversight mechanisms, (3) inclusive symbols and language, (4) stakeholder involvement, and (5) intergenerational initiatives. Findings highlight systemic, socio-structural, and psychological challenges in fostering inclusive environments. Despite these barriers, facilitators indicate that meaningful change is achievable. Strengthening organizational strategies and fostering sustainable institutional change are critical to building more equitable communities. Addressing these issues requires targeted policy interventions, cross-sector collaboration, and long-term investment in inclusive practices to ensure older LGBT+ people and those with LD are fully recognized and supported in society.

